# The *Pax* gene family: Highlights from cephalopods

**DOI:** 10.1371/journal.pone.0172719

**Published:** 2017-03-02

**Authors:** Sandra Navet, Auxane Buresi, Sébastien Baratte, Aude Andouche, Laure Bonnaud-Ponticelli, Yann Bassaglia

**Affiliations:** 1 UMR BOREA MNHN/CNRS7208/IRD207/UPMC/UCN/UA, Muséum National d'Histoire Naturelle, Sorbonne Universités, Paris, France; 2 Univ. Paris Sorbonne-ESPE, Sorbonne Universités, Paris, France; 3 Univ. Paris Est Créteil-Val de Marne, Créteil, France; Laboratoire de Biologie du Développement de Villefranche-sur-Mer, FRANCE

## Abstract

*Pax* genes play important roles in Metazoan development. Their evolution has been extensively studied but Lophotrochozoa are usually omitted. We addressed the question of *Pax* paralog diversity in Lophotrochozoa by a thorough review of available databases. The existence of six Pax families (Pax1/9, Pax2/5/8, Pax3/7, Pax4/6, Paxβ, PoxNeuro) was confirmed and the lophotrochozoan Paxβ subfamily was further characterized. Contrary to the pattern reported in chordates, the Pax2/5/8 family is devoid of homeodomain in Lophotrochozoa. Expression patterns of the three main *pax* classes (*pax2/5/8*, *pax3/7*, *pax4/6*) during *Sepia officinalis* development showed that Pax roles taken as ancestral and common in metazoans are modified in *S*. *officinalis*, most likely due to either the morphological specificities of cephalopods or to their direct development. Some expected expression patterns were missing (e.g. *pax6* in the developing retina), and some expressions in unexpected tissues have been found (e.g. *pax2/5/8* in dermal tissue and in gills). This study underlines the diversity and functional plasticity of *Pax* genes and illustrates the difficulty of using probable gene homology as strict indicator of homology between biological structures.

## Introduction

Pax proteins belong to a family of transcription factors playing important roles in development of metazoans, from early specification of cell fate to body patterning through morphogenesis of various tissues and organs [1,2]. The evolution of *Pax* genes has been extensively studied and the availability of genomes has allowed for the identification of *Pax* genes in nearly 200 species of chordates in which duplication and subfunctionalization may have occurred several times [3]. *Pax* genes have also been identified in non-chordates, but no exhaustive study of *Pax* gene evolution has been conducted regarding these clades. In fact lophotrochozoans, which comprise approximately 30% of all animal species, are usually omitted in studies of *Pax* gene evolution (see [4] as a recent example).

Pax proteins are characterized by the presence of a DNA-binding domain of 128 amino-acids, referred to as the paired domain (PRD) (review in [5]). Other conserved domains may be present in the sequence of Pax proteins after the PRD: an octapeptide motif (OM) and part or all of a paired-type homeobox DNA-binding domain (HD). A common ancestor (“Ur-pax-gene”) containing the three domains would have led to the “classical” five classes of *Pax* genes recognized in Ecdysozoa and Chordata (*pox-neuro*, *pax4/6*, *pax2/5/8*, *pax1/9* and *pax3/7*) by gene duplications and subsequent deletions before the divergence of Protostomia and Deuterostomia [6–10]. Moreover, a sixth *Pax* clade (*pax-a/β*) has recently been proposed by some authors ([11–13]) and the status of a seventh class (*eyg*) remains unclear [14]. In addition, further gene or genome duplication events leading to different *Pax* paralogs, as well as alternative splicing, are known to generate numerous *Pax* isoforms in chordate (e.g. [15–17]). No basal genome duplication has been demonstrated in Lophotrochozoa and they are thought to use a restricted repertoire of Pax proteins. Nevertheless some *Pax* isoforms have been characterized. Each of *pax*6 [18], *pax* 3/7 [19] and *pax β* [12] have two isoforms in *Helobdella robusta* (Annelida). Five isoforms of *pax*6 resulting from alternative splicing without genome duplication have been characterized in *Idiosepius paradoxus* (Mollusca Cephalopoda) embryos [20]. A recent article has extensively studied the evolution of *pax2/5/8* among molluscs [21] but no extensive search has addressed the question of *Pax* paralog diversity in Lophotrochozoa. A first objective of this paper is to thoroughly characterize the set of *Pax* genes in Lophotrochozoa.

**Fig 1:**
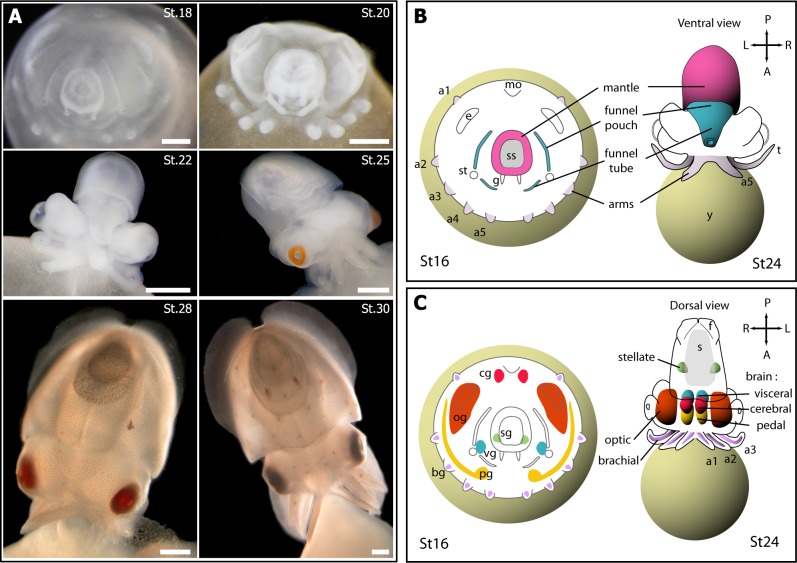
Development of *Sepia officinalis* and localisation of main nervous structures (after [38], modified). **(A) Organogenesis**. **St18**: Plane phase (Stage 14–18) Stage 18, aboral view; the embryo is “disc-shaped” on the surface of yolk. **St20, St22**: Extension phase (Stages 19–22). St20: Stage 20, aboral view; the shell sac is closed and the two funnel tube elements grow. St22: Stage 22, ventral view; the funnel tube is formed. **St25, St28, St30**: Growth phase (Stages 23–30), dorsal view. St25: Stage 25; the shell begins to form, eyes are clearly coloured. St28: Stage 28; skin is clearly coloured. St30: Stage 30, just before hatching. (**B) Muscular system**: the mantle, the two elements of the funnel (funnel pouches and funnel tube) and arms, essential for locomotion, are coloured. (**C) Nervous system**: at stage 16, nervous system is composed of sparse ganglia. Cerebroid ganglia (red) will constitute the dorsal supraoesophageal mass of the brain, pedal (yellow) and visceral (blue) ganglia will constitute the anterior/median and posterior (respectively) sub-oesophageal mass of the brain (depicted in stage 24 dorsal view). Optic ganglia (orange) will constitute optic lobes; with the brain, they constitute the central nervous system. Stellate ganglia (green), located symmetrically on the internal side of the mantle and brachial ganglia (one by arm, which develops into a cord inside the arm) belong to the peripheral nervous system. For more information about development see [53]) a1, a2, a3, a4, a5: arms 1 to 5; bg, brachial ganglia; cg, cerebroid ganglia; e: eye; g: gill; mo: mouth; og, optic ganglia; pg, pedal ganglia; s, shell; st, statocyst; sg, stellate ganglia; ss: shell sac; t: tentacle; vg, visceral ganglia; y: yolk. Orientation: A(nterior)–P(osterior), L(eft)–R(ight). Scale bar: 1 mm.

The diversity of *Pax* molecular family is assumed to explain the high functional diversity of Pax proteins [10,22] however there is no unique developmental function for each Pax despite the highly conserved general structure of *Pax* genes [23,24]. In lophotrochozoan species studied so far (Platyhelminthes [25,26], Annelida [18,19,23,27,28], Mollusca [21,29–34], Brachiopoda [35,36], Nemertea [37]), expression patterns of each *Pax* gene suggest conserved and consistent roles for *pax*3/7 in nervous system development, *pax*2/5/8 in sensory structure formation and *pax*6 in eye morphogenesis. However, our different on-going studies regarding *Sepia officinalis Pax* genes during development [33,34,38–40] have called into question the consistency between *Pax* gene structure, expression and role in cephalopod *Pax* gene family. Thus, a second objective of this paper is to complete our previous studies on the expression patterns of *Pax* genes in the cuttlefish *Sepia officinalis* and to compare these expressions with other Lophotrochozoa. Expression patterns of the three main *Pax* classes during development show that *Pax* roles, taken as ancestral (e.g. [21]) and common in metazoans, are modified in *S*. *officinalis*. Changes of Pax roles we observed could be linked to an unusual body plan [41], a direct development and numerous morphological novelties which are specific to Cephalopoda, such as muscular and nervous structures related to specific behaviors, locomotion and cognitive abilities (see Fig 1). Comparison of gene expression patterns between metazoans is often used as a footprint for homology in evo-devo studies, disregarding body plan or developmental differences [42]. Therefore, our results remind that such a paradigm should always be used carefully.

## Materials and methods

### Sequence analysis

Putative Pax proteins were searched for using protein databases (Uniprot, NCBI protein) and accessible draft genomes (*Octopus bimaculoides*: metazome website https://metazome.jgi.doe.gov/pz/portal.html#!info?alias=Org_Obimaculoides_er, *Pinctada fucata*: OIST marinegenomics website http://marinegenomics.oist.jp/pearl/viewer/info?project_id=20 [43]), transcriptome (our EST bank, [44]) or proteome (*Mytilus galloprovincialis* [45]) to construct a set of sequences restricted to the lophotrochozoan clade. Alignments were done using Jalview2 [46]. Phylogenetic analysis was performed on the “phylogeny.fr” platform [47] accessible at http://www.phylogeny.fr. After alignment, ambiguous regions (i.e. containing gaps and/or poorly aligned) were removed with Gblocks [48] using the following parameters: minimum length of a block after gap cleaning: 10, no gap positions allowed in the final alignment, all segments with contiguous non-conserved positions bigger than 8 rejected, minimum number of sequences for a flank position: 85%. The phylogenetic tree was reconstructed using the maximum likelihood method implemented in the PhyML (v3.1/3.0 aLRT) program [49]. The default substitution model was selected assuming an estimated proportion of invariant sites (of 0.088) and 4 gamma-distributed rate categories to account for rate heterogeneity across sites. The gamma shape parameter was estimated directly from the data (gamma = 0.716). Reliability for internal branch was assessed using the aLRT test (SH-Like) [50] or using the bootstrapping method (100 bootstrap replicates); all branches with support <50% were collapsed. Graphical representation and edition of the phylogenetic tree were performed with TreeDyn v198.3 [51].

### Expression patterns

*Sepia officinalis* eggs were obtained from captive females (maintained in the biological station of Luc-sur-Mer, France) and maintained at 18°C in oxygenated seawater in the laboratory. The experimental procedures were carried out in strict compliance with the European Communities Council Directive (86/609/EEC) and followed the French legislation requirements (decree 87/848) regarding the care and use of laboratory animals, under the control of the ethics committee of the Muséum National d'Histoire Naturelle (“Comité Cuvier-68”). All efforts were made to minimize animal suffering and to reduce the number of animals used.

Embryos were sampled daily to assemble a complete collection of morphological stages from stage 14 (beginning of organogenesis) according to the developmental table established by Lemaire [52] and revised by Boletzky et al. [53]. The dark pigmented egg capsule and the chorion were removed in seawater. Embryos were maintained on ice in seawater until lethargy. They were then fixed and processed for *in situ* hybridization (ISH).

RNA extraction, cDNA synthesis and gene cloning of *Sof-pax3/7* and *Sof-pax6* have been described previously [33,34,54]. For *Sof-pax2/5/*8, the primers F-5’- ACCTAACCACAGCGTACCGT-3’ (DLTTAYR) and R-5’-GACCATGTTTGCCTGGGAGA-3’ (TMFAWE) were used to obtain a 420 bp fragment. The primers were designed in order to target each gene but without discrimination of their potential splicing variants: in addition to specific regions of *pax6*, *pax3/7* and *pax2/5/8* genes, a large part of the conserved Paired Domain was included in all probes (see S1 Primers for primer positions). Procedures for cloning, RNA probes synthesis, whole-mount ISH and sectioning have been described previously [34]. Embryos were observed with a Leica M16 2F binocular stereomicroscope and a Leica DMLB compound microscope. Maximum intensity projections were generated using ImageJ (http://rsbweb.nih.gov/ij/). All images were adjusted for contrast and brightness and assembled into plates using Adobe Photoshop 8 or CS4 (Adobe, San Jose, CA, USA).

## Results

### Pax alignments and Pax family in Lophotrochozoa

After an extensive search, a set of 216 putative Pax protein sequences restricted to the lophotrochozoan clade was compiled (S1 Table). As expected, the paired domain (PRD) signature of Pax proteins was highly conserved. A first alignment was made by automatic analysis based on the PRD domain but the general alignment of all sequences was manually obtained (S1 Alignment) using the PRD, OM (Octapeptide motif) and HD (Homeodomain) as visual guides.

**Fig 2:**
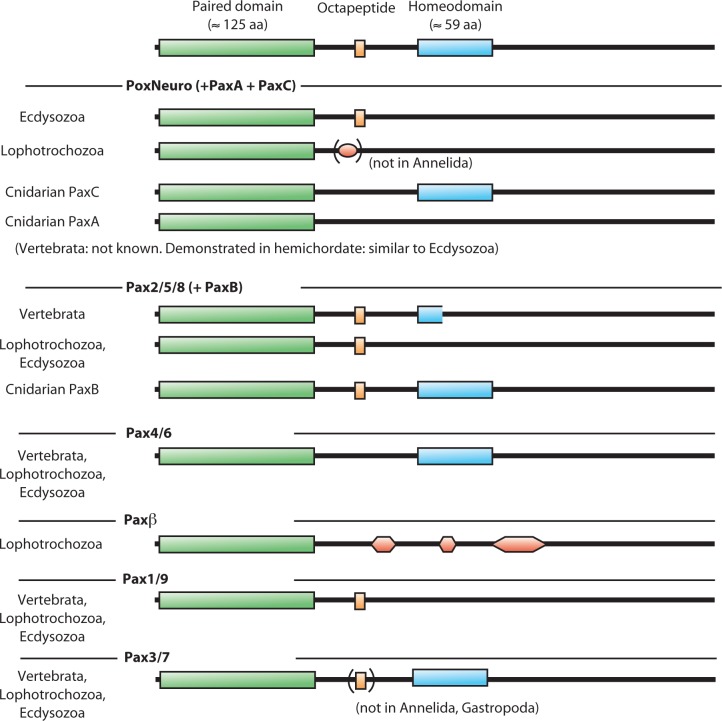
Domain composition of Pax proteins. Data from [8,9,13] and this work. The known lophotrochozoan sequences present some distinctive features: no OP in *PoxNeuro*, no HD in *Pax2/5/8*, presence of *Paxβ* with characteristic signals.

**Fig 3:**
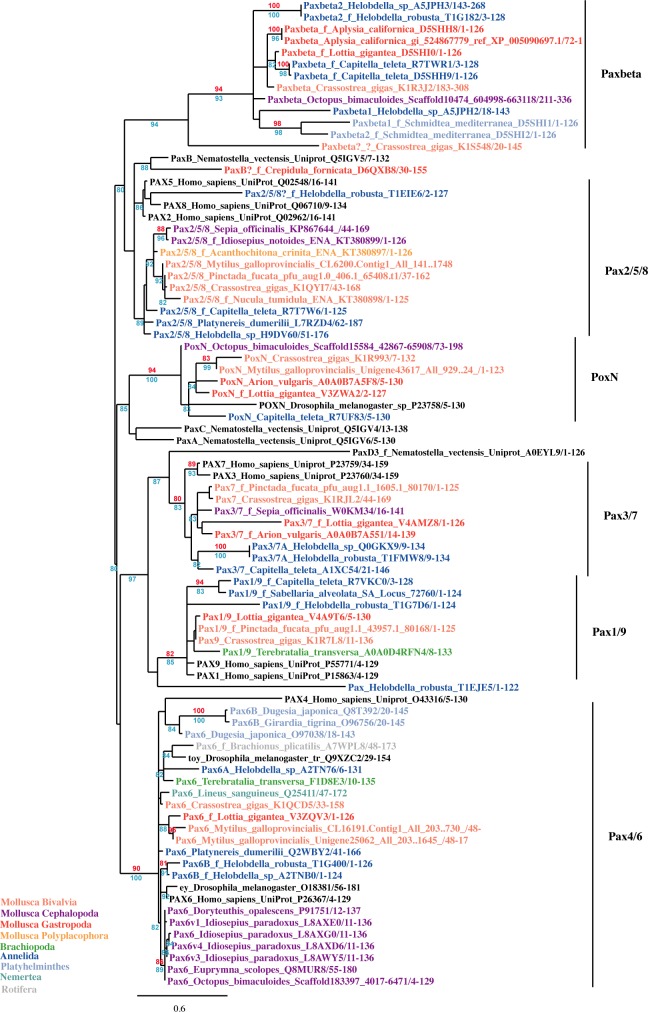
Unrooted phylogenetic tree of lophotrochozoan *Pax* proteins. All branches with support <50% were collapsed. The results of approximate Likelihood-Ratio Test (SH-Like) (blue) and bootstrap (red) are indicated if >80%. The sequences used and their reference numbers are given in S1 Table. The names used in the tree are our proposed identifications of these sequences if different from original annotations (see the differences between submitted name and proposed name in Table 1 and S1 Table).

The PRD was used to construct phylogenetic trees of the lophotrochozoan Pax proteins. Human, *Drosophila* and *Nematostella* sequences were included to facilitate the identification of the classes of proteins obtained after phylogenetic reconstruction. The sets used in these analyses were obtained by elimination of redundant sequences (e.g. sequences from the same species containing 100% identity in the PRD) and sequences lacking important parts or whole PRD (e.g. 93 partial PRD sequences from various Cephalopoda and all putative *eyg* sequences). Depending on the stringency of this elimination, the procedure led to sets from 71 to 65 lophotrochozoan PRD sequences, which could be analysed on 108 to 121 positions (respectively). The phylogenetic trees obtained with these different sets were comparable and confirmed the presence of the 6 Pax families in Lophotrochozoa proposed by Schmerer et al. [12] (Fig 2). One representative phylogenetic tree is presented (Fig 3). The following description of each of these families is based on the alignment and supported by the phylogeny. 56 sequences that were previously non- or mis-identified are listed in Table 1.

**Table 1:** Previously non- or mis-identified lophotrochozoan Pax protein sequences.

Accession	Submitted name	Proposed name	species	Classification
T1EJE5	Uncharacterized protein	**Pax**	Helobdella_robusta	Annelida_Clitellata
T1G7D6	Uncharacterized protein	**Pax1/9(f)**	Helobdella_robusta	Annelida_Clitellata
T1EIE6	Uncharacterized protein	**Pax2/5/8?(f)**	Helobdella_robusta	Annelida_Clitellata
T1EH18	Uncharacterized protein	**Pax2/5/8(f)**	Helobdella_robusta	Annelida_Clitellata
T1FMW8	Uncharacterized protein	**Pax3/7A**	Helobdella_robusta	Annelida_Clitellata
T1G8F8	Uncharacterized protein	**Pax3/7B(f)**	Helobdella_robusta	Annelida_Clitellata
T1F6U6	Uncharacterized protein	**Pax6A**	Helobdella_robusta	Annelida_Clitellata
T1G400	Uncharacterized protein	**Pax6B(f)**	Helobdella_robusta	Annelida_Clitellata
T1EHA5	Uncharacterized protein	**Paxβ1(f)**	Helobdella_robusta	Annelida_Clitellata
T1G182	Uncharacterized protein	**Paxβ2(f)**	Helobdella_robusta	Annelida_Clitellata
R7VKC0	Uncharacterized protein	**Pax1/9(f)**	Capitella_teleta	Annelida_Polychaeta
R7T7W6	Uncharacterized protein	**Pax2/5/8(f)**	Capitella_teleta	Annelida_Polychaeta
R7TKD0	Uncharacterized protein	**Pax3/7(f)**	Capitella_teleta	Annelida_Polychaeta
R7TWR1	Uncharacterized protein	**Paxβ(f)**	Capitella_teleta	Annelida_Polychaeta
R7UF83	Uncharacterized protein	**PoxN**	Capitella_teleta	Annelida_Polychaeta
K1QWY6	Paired box protein Pax-6	**eyg**	Crassostrea_gigas	Mollusca_Bivalvia
K1QYI7	Paired box protein Pax-2-A	**Pax2/5/8**	Crassostrea_gigas	Mollusca_Bivalvia
K1R3J2	Paired box protein Pax-2-A	**Paxβ**	Crassostrea_gigas	Mollusca_Bivalvia
K1S548	Paired box protein Pax-6	Paxβ(?)	Crassostrea_gigas	Mollusca_Bivalvia
K1R993	Paired box protein Pax-8	**PoxN**	Crassostrea_gigas	Mollusca_Bivalvia
Unigene67849_All_[769..2]	Unigene67849_All_[769..2]	**Pax1/9(f)**	Mytilus_galloprovincialis	Mollusca_Bivalvia
CL6200.Contig1_All_[141..1748]	CL6200.Contig1_All_[141..1748]	**Pax2/5/8**	Mytilus_galloprovincialis	Mollusca_Bivalvia
CL6200.Contig2_All_[141..1640]	CL6200.Contig2_All_[141..1640]	**Pax2/5/8**	Mytilus_galloprovincialis	Mollusca_Bivalvia
CL6200.Contig3_All_[308..1978]	CL6200.Contig3_All_[308..1978]	**Pax2/5/8**	Mytilus_galloprovincialis	Mollusca_Bivalvia
CL6200.Contig4_All_[308..2086]	CL6200.Contig4_All_[308..2086]	**Pax2/5/8**	Mytilus_galloprovincialis	Mollusca_Bivalvia
CL6200.Contig5_All_[308..1885]	CL6200.Contig5_All_[308..1885]	**Pax2/5/8**	Mytilus_galloprovincialis	Mollusca_Bivalvia
CL6200.Contig6_All_[308..1993]	CL6200.Contig6_All_[308..1993]	**Pax2/5/8**	Mytilus_galloprovincialis	Mollusca_Bivalvia
Unigene61312_All_[208..2]	Unigene61312_All_[208..2]	**Pax3/7(f)**	Mytilus_galloprovincialis	Mollusca_Bivalvia
CL16191.Contig1_All_[203..730]	CL16191.Contig1_All_[203..730]	**Pax6**	Mytilus_galloprovincialis	Mollusca_Bivalvia
Unigene25062_All_[203..1645]	Unigene25062_All_[203..1645]	**Pax6**	Mytilus_galloprovincialis	Mollusca_Bivalvia
CL16191.Contig2_All_[1..435]	CL16191.Contig2_All_[1..435]	**Pax6(f)**	Mytilus_galloprovincialis	Mollusca_Bivalvia
Unigene43617_All_[929..24]	Unigene43617_All_[929..24]	**PoxN**	Mytilus_galloprovincialis	Mollusca_Bivalvia
Scaffold15272:139788–157863	Ocbimv22007526m.p	**Pax2/5/8**	Octopus_bimaculoides	Mollusca_Cephalopoda
Scaffold183397:4017–6471	Ocbimv22011111m.p	**Pax6**	Octopus_bimaculoides	Mollusca_Cephalopoda
Scaffold17697:31829–33450	Ocbimv22010462m.p	**Pax9**	Octopus_bimaculoides	Mollusca_Cephalopoda
Scaffold10474:604998–663118	Ocbimv22000807m.p	**Paxβ**	Octopus_bimaculoides	Mollusca_Cephalopoda
Scaffold15584:42867–65908	Ocbimv22007901m.p	**PoxN**	Octopus_bimaculoides	Mollusca_Cephalopoda
gi|524867779|ref|XP_005090697.1	PREDICTED: mucin-5AC	**Paxβ**	Aplysia_californica	Mollusca_Gastropoda
A0A0B6YZY1	Uncharacterized protein (Fragment) GN = ORF42948	**Pax2/5/8?(f)**	Arion_vulgaris	Mollusca_Gastropoda
A0A0B7A551	Uncharacterized protein (Fragment) GN = ORF96941	**Pax3/7(f)**	Arion_vulgaris	Mollusca_Gastropoda
A0A0B7A6X2	Uncharacterized protein (Fragment) GN = ORF96938	**Pax3/7(f)**	Arion_vulgaris	Mollusca_Gastropoda
A0A0B7A6Y7	Uncharacterized protein (Fragment) GN = ORF96935	**Pax3/7(f)**	Arion_vulgaris	Mollusca_Gastropoda
A0A0B6Y326	Uncharacterized protein (Fragment) GN = ORF11503	**Pax6(f)**	Arion_vulgaris	Mollusca_Gastropoda
A0A0B6YFJ8	Uncharacterized protein (Fragment) GN = ORF24120	**Pax6(f)**	Arion_vulgaris	Mollusca_Gastropoda
A0A0B7A5F8	Uncharacterized protein	**PoxN**	Arion_vulgaris	Mollusca_Gastropoda
gi|908452192|ref|XP_013082659.1	PREDICTED: mucin-5AC-like	**Paxβlike**	Biomphalaria_glabrata	Mollusca_Gastropoda
A0A0B6VJL1	Paired box 6 protein	**eyg**	Lottia_gigantea	Mollusca_Gastropoda
V4A9T6	Uncharacterized protein	**Pax1/9**	Lottia_gigantea	Mollusca_Gastropoda
V3ZI38	Uncharacterized protein	**Pax2/5/8(f)**	Lottia_gigantea	Mollusca_Gastropoda
V4AMZ8	Uncharacterized protein	**Pax3/7(f)**	Lottia_gigantea	Mollusca_Gastropoda
V3ZQV3	Uncharacterized protein	**Pax6(f)**	Lottia_gigantea	Mollusca_Gastropoda
Contig4275(2463977–2470937)	LgGsHFWreduced.5213	**Paxβ**	Lottia_gigantea	Mollusca_Gastropoda
V4B0D1	Uncharacterized protein	**Paxβ(f)**	Lottia_gigantea	Mollusca_Gastropoda
V3ZWA2	Uncharacterized protein	**PoxN(f)**	Lottia_gigantea	Mollusca_Gastropoda
Q25411	Pax6-like protein	**Pax6**	Lineus_sanguineus	Nemertea_Anopla
O96756	DtPax-6 protein	**Pax6B**	Girardia_tigrina	Platyhelminthes_Rhabditophora

Accession: accession number or genome reference. Submitted name: name found in databases. Proposed name: our interpretation (grey background if different from a submitted name); (f) denote a fragment. Sequences are included in S1 Table.

#### PoxNeuro

To our knowledge, the only PoxNeuro already signalled in molluscs before this study was *Pon* in *Pinctada fucata* [55]. Our phylogenetic analysis demonstrated the existence of other PoxNeuro, non- or mis-identified (see Table 1 and S1 Table), in molluscan clades (Gastropoda, Bivalvia and Cephalopoda), as well as in Annelida.

These sequences showed a tribasic signal in the first helix of the PRD and insertions corresponding to the known exon2 / exon3 junction in *Drosophila* (KPK**Q**VAT) between the N-ter (PAI domain) and C-ter (RED domain) domains of the PRD. The HD was absent. By contrast with PoxNeuro from Ecdysozoa or the hemichordate *Saccoglossum kowalevskii* ([13,56], and our alignments not shown), no OM was clearly detected in Lophotrochozoa. However, a commonly found [VI]PGLSYP[KR][IL]V motif was present at least in Mollusca after the PRD.

#### Pax2/5/8

The *Pax2/5/8* group was well identified but moderately supported in phylogenetic analysis. Contrary to the structure described in chordate, no partial HD could be identified in any lophotrochozoan Pax2/5/8. By contrast, a clear octapeptide (Y[TS]IX_2_ILG) was present, followed by a quadribasic/diacid signal already underlined in chordate (KR-rich region of [57]).

The transcriptome of *Mytilus galloprovincialis* revealed 6 isoforms of *Pax2/5/8*. The N-ter region flanking the PRD suggested two different groups of sequences, which may reflect the existence of two different genes and alternative splicing. By contrast, the existence of different isoforms of Pax2/5/8 in Annelida remained elusive. The *Helobdella sp*. complete sequence H9DV60 revealed two N-rich regions, reminiscent of the description of Paxβ by Schmerer et al. [12]. These regions surrounded a clear octapeptide but without the KRKR signal of Pax2/5/8. Nevertheless, this sequence was clearly assigned to the *Pax2/5/8* group in accordance with its gene organisation description [28]. Unfortunately, the *Helobdella robusta* Pax2/5/8 sequence fragment T1EH18, flagged as « complete » but with « non-terminal residues » in Uniprot, does not cover the OM zone. The *Helobdella robusta* sequence T1EIE6 is quite different (% identity = 72% in the PRD) and appeared linked to different groups, depending on the set analysed.

#### Pax4/6

The Pax4/6 subfamily was clearly identified. These sequences displayed a canonical structure (PRD, no octapeptide, HD), including the characteristic MDKL pattern between PRD and HD [37].

Two Pax6 isoforms are known in Planaria, one of which (Pax6B) described as specific to this clade [58]. We did not detect any non-planarian homologue to the planarian B isoform which was the only Pax6 devoid of the MDKL motif. Noteworthy, the two planarian isoforms constituted a well-supported clade in phylogenetic analysis using the paired domain, in accordance with the results of Quigley et al. [18].

The transcriptome of *Mytilus galloprovincialis* contained at least 2 forms of *Pax6*: a long canonical isoform and a short isoform reduced to the paired zone. The only *Pax6* detected in the genome of *Octopus bimaculoides* was lacking a homeodomain, although complete Pax6 are already known in other cephalopods [20]. The *Crassostrea gigas* Pax6 showed an insertion in the C-ter part (RED domain) of the PRD, reminiscent of the human Pax6-5a isoform which contains an insertion in the PAI domain thought to modulate the interaction between the Pax transcription factors and their DNA consensus sequence [59–62].

Two sequences previously identified as Pax6 (*Lottia gigantea* A0A0B6VJL1 and *Crassostrea gigas* K1QWY6) were in fact identified as *eyegone* homologues. This gene was also present in *Pinctada* genome: two sequences have already been signalled, most likely reflecting two allelic copies [55].

#### Pax beta

A Pax beta subfamily was present in phylogenetic analysis with a good support. This subfamily has been described as lophotrochozoan-specific with two forms known in Annelida (*Paxβ1*, *Paxβ2*), corresponding to two genes [12]. *Paxβ* complete sequences were found in *Octopus bimaculatus* (Ocbimv22000807m.p), *Lottia gigantea* (LgGsHFWreduced.5213) and *Crassostrea gigas* (K1R3J2), the later misidentified as Pax-2-A by automatic annotation. These molluscan sequences did not show the N-rich regions mentioned in *Helobdella* sequences [12]. Their organization included a PRD followed by a very long and variable region, in which three conserved motifs could be detected: (YDY[NS]LPDRGL), (PLDLS), and (Y[ED][RK]N[LVM]L[LI]FGD[SNQ]E[IVL]EI[MI]SVGKX[KR]W[IV][VI]RNEX[DE]L). None of these patterns was found in databases and their significance remains unknown. Using the longest motif in a Blast search led to the identification of a very short sequence of *Arion vulgaris* (A0A0B6XZN0), a complete sequence of *Aplysia californica* (XP_005090697.1, identified as mucin-5AC) and a *Biomphalaria glabrata* sequence showing all the conserved motifs but lacking the PRD (also identified as mucin-5AC like). Curiously, the corresponding *Aplysia* mRNA sequence was previously annotated as *Pax beta* (from automatic annotation) and this sequence was included in our set as a *bona fide* Paxβ. The identification of the *Biomphalaria* sequence remains questionable and therefore, we included it in our set as a Paxβ-like.

The *Crassostrea gigas* sequence K1S548, automatically annotated as Pax6, was grouped with the Paxβ with a good support (aLRT) despite its different PRD sequence (% Identity with K1R3J2 = 58%), a typical *Pax6*-type PRD N-ter sequence (SGVNQL) and the absence of the three motifs already signalled. This sequence may be the first example of *Paxβ* duplication outside of the Annelida clade. Interestingly, this sequence contains a quadribasic/quadriacid (K^208^RKHEDED) signal down to the PRD domain. This is reminiscent of Pax2/5/8 and consistent with proximity between *Paxβ* and *Pax2/5/8* as suggested by Schmerer et al. [12]. However, this proximity was only weakly supported by our phylogenetic analysis.

#### Pax1/9

The *Pax1/9* subfamily contained a clear octapeptide (H[ST]V[ST][DN][IL]LG) and no HD. In this group, the status of *Helobdella robusta* T1EJE5, a fragment flagged as « complete » but with « non-terminal residues » in Uniprot, was unclear. This fragment was poorly similar (% identity = 60) to a clear *Helobdella robusta* Pax1/9 (T1G7D6). Depending of the set of sequences used in the phylogenetic analysis, it was included or not in the *Pax1/9* group. Surprisingly, we failed to detect or clone *Pax1/9* from *S*. *officinalis* whereas *bona fide Pax1/9* sequences from *Octopus* were found in databases (not included in our phylogenetic analysis due to their incomplete PRD). Further exploration is needed to resolve this discrepancy.

#### Pax3/7

The *Pax3/7* subfamily was also well supported, but the structures of these proteins were variable. The PRD and HD were present in all sequences, but the octapeptide was only obvious in cephalopods and in one Bivalvia (*Crassostrea gigas*). No clear homologue of the octapeptide could be evidenced in Gastropoda, Annelida and in the Bivalvia *Pinctada fucata*.

Two forms of Pax3/7, Pax3/7A and Pax3/7B, have been described in *Helobdella sp*. (Austin), corresponding to two different genes [19]. They were also identified in *Helobdella robusta* but no evidence of Pax3/7B was found in other species including the Annelida *Capitella teleta*, implying that the duplication suggested by Woodruff et al. [19] was restricted to an Annelida sub-clade.

Three different *pax3/7* sequences have been identified in *Arion vulgaris*, issued from the same contig but with different cDNA sequences. Theses sequences showed the same insertion in PRD, possibly reflecting a particular structure of these genes.

### *Pax* genes expression patterns in *Sepia officinalis*

We compared the expression patterns of *pax6*, *pax2/5/8* and *pax3/7* based on our previous results [33,34,39,40] and new ones obtained during *Sepia* development. Organogenesis of cephalopods takes place in three main phases (Fig 1A) and is only briefly described here (see [53] for details). First, the animal pole of the embryo is shaped as a disk in which ectoderm and mesendoderm differentiate and organs start delineating: the arms at the periphery and the mantle at the centre of the disc (stage 14 to 18, see Fig 1). In a second phase (stage 19 to 21), the animal pole increases its volume and starts elongating: the arm crown becomes anterior (it surrounds the mouth) and the mantle acquires its definitive posterior position (the anus is located in the mantle cavity). Between both extremities, most of the nervous ganglia concentrate inside the future head and the muscular funnel surrounds the mantle border (Fig 1). In the third phase, the embryo acquires the definitive juvenile shape. Inside the head, nervous ganglia develop as brain lobes and optic lobes. The development of muscular and nervous territories in *S*. *officinalis* are summed up in Fig 1B and 1C.

#### *Sof-pax 6* expression

**Fig 4:**
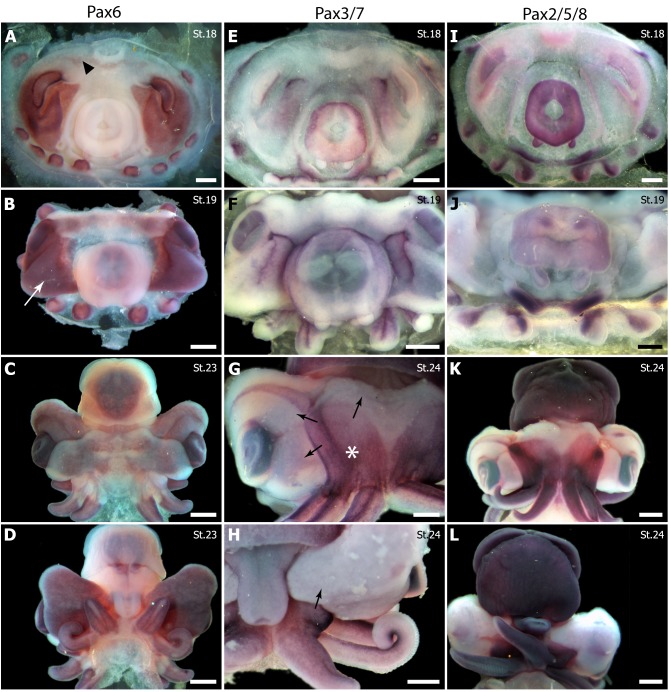
*Pax* gene expression pattern by *in toto* ISH during *Sepia officinalis* development. (A-D) *Pax6*. (E-H) *Pax3/7*. (I-L) Pax2/5/8. Stages are indicated by St#; C,G,K, dorsal view; D,H,L, ventral view. Scale: 1 mm. (A) A large optic area and the five arms are strongly *Pax6*-positive, a light expression is observed in cerebroid ganglia (arrowhead). (B) All the tissues surrounding the eye express *Pax6* including cheek (white arrow). (C/D) The distal part of the arms is *Pax6*-positive. Note the artefact in the shell area. (E/F) Aboral side of the arms, anterior part of the mantle and funnel tube elements express *Pax3/7*. (G/H) Dorsal arm pillars (asterisk) and aboral side of the arms are *Pax3/7*-positive. Black arrows denote the future extension of arm pillars. (I/J) Mantle, gills, funnel tube elements and oral side of the arms are *Pax2/5/8*-positive. (K/L) Mantle, fins and arms express *Pax2/5/8*.

Expression of *Sepia officinalis pax6* (*Sof-pax6*) was observed from early stages (stage 14) and throughout the development. This expression was apparently restricted to structures of ectodermal origin. As organogenesis progresses, *Sof-pax*6 was strongly expressed in a large area from the optical region to statocystes from stage 15 to 18 (Fig 4A). As in other cephalopods (*Loligo opalescens* [29]*; Euprymna scolopes* [32]), *pax6* expression in *S*. *officinalis* was observed in cerebroid and optic ganglia, from stage 19 (Fig 4A and 4B and [33]). At stage 23, optic lobes (former optic ganglia) were prominent and expressed *Sof-pax*6, as did the lateral supraoesophageal mass and the sub-pedunculate tissue of the cheek (Fig 4D), a neuro-endocrine tissue coming from the center of optic lobe [63]. *Sof-pax6* was not expressed in pedal and visceral ganglia, two ganglia leading to the suboesophageal mass.

**Fig 5:**
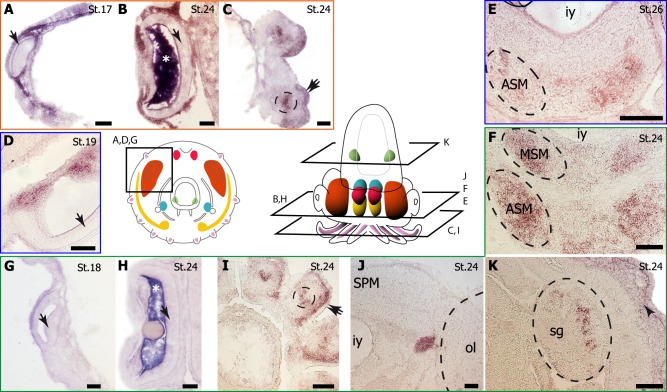
*Pax* gene expression in eye and the nervous system. (A-C) *Pax6*; (D,E) *Pax3/7*; (F-K) *Pax2/5/8*. Stages are indicated by St#. Note the artefact in vitreous humour in the eye (asterisk). E, F and J are similar cutting planes, E, being the most anterior. Scale: 150 μm. The retina (arrow) is devoid of *Pax6* (A,B), *Pax3/7* (D) and *Pax2/5/8* (G, H) expression although surrounding tissues express *Pax6* and/or *Pax3/7*. At stage 24, the brachial cord (circle) expresses *Pax6* (C) and *Pax2/5/8* (I). The arm epidermis also expresses *Pax6* but not *Pax2/5/8* (double-arrow). The anterior suboesophageal mass expresses *Pax3/7* (ASM in E) and *Pax2/5/8* (ASM in F). The middle suboesophageal mass expresses *Pax2/5/8* (MSM in F). The optic tractus (J) and the stellate ganglia (sg in K) express *Pax2/5/8*, as the dermal part of the skin (arrowhead). iy: internal yolk sac. ol: optic lobe. SPM: supraoesophageal mass.

**Fig 6:**
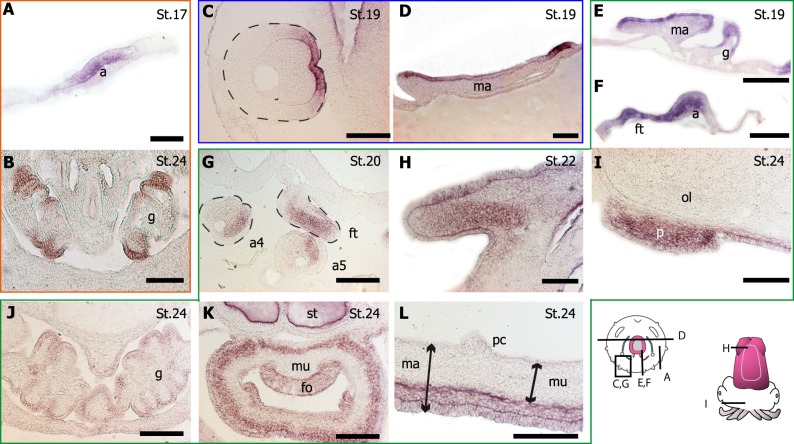
*Pax* gene expression in non neuronal structures. (A, B) *Pax6*; (C, D) *Pax3/7*; (E-L) *Pax2/5/8*. Stages are indicated by St#. Note the artefact in statocyst (st) in K. Scale 150 μm, except E, F, G: 300 μm. **Pax expression in arm** (a): *Pax6* (A. See also Fig 5C), *Pax3/7* (C) and *Pax2/5/8* (F, G a4 and a5. See also Fig 5I). **Pax expression in mantle** (ma): *Pax3/7* (D) and *Pax2/5/8* (E, L). **Pax expression in gill** (g): *Pax6* (B) and *Pax2/5/8* (E, J). **Dynamic of *Pax2/5/8* expression in funnel tube** (ft): compare F, G and K (fo: funnel organ). Pax2/5/8 is also expressed in fin (H), arm pillars (p) (I). ol: optic lobe. mu: muscle. pc: pallial cavity. ss: shell sac.

*Sof-pax6* was expressed from stage 17 in arm epidermis (Fig 4A, Fig 5C, Fig 6A). Later, *Sof-pax6* was expressed in the distal part of the arms with a strict and straight limit (Fig 4C). From stage 23, *Sof-pax6* was also expressed in intrabrachial nervous cords (Fig 5C). Finally, an expression of *Sof-pax6* was observed in gill epithelium at stage 24, where *Sof-pax2/5/8* is also detected (Fig 6B and 6J) (see below).

#### *Sof-pax3/7* expression

We pushed forward with our previous *Sof-pax3/7* expression study on sensory structures in *S*. *officinalis* [34]. *Sof-pax3/7* is expressed as early as stage 16 in most parts of epidermal tissues. At stage 19, *Sof-pax3/7* was expressed in skin epithelium (ectodermal cells) of the mantle and arms (Fig 4E, Fig 6C and 6D). From stage 23/24 to 27, *Sof-pax*3/7 expression was clearly observed on the arm pillars area that will extend and cover the early cephalic tissue, forming the secondary cornea at the level of the eyes (Fig 4G and 4H).

From stage 22, *Sof-pax3/7* was also expressed in the nervous system, first in pedal ganglia and later in the anterior part of the suboesophageal mass (formed by these ganglia), more specifically in pre-brachial and brachial lobes (Fig 5E) involved in arm control [64]. No expression of *Sof-pax3/7* was detected in the supraoeophageal part of the brain, in the buccal ganglia or in optic lobes. An expression of *Sof-Pax3/7* was observed from stage 16 to 20 in the upper side of optic vesicle corresponding to lentigenic tissues (Fig 5D). This expression disappeared at the same time the lens is forming.

There was no expression of *Sof-pax3/7* in mesodermal tissues, such as gills and muscles in the mantle or the funnel. By contrast, *Sof-pax3/7* expression was detected in the epidermal funnel organ, a prominent structure producing mucus [65,66], which also expressed *So-pax2/5/8* (see below).

#### *Sof-pax2/5/8* expression

In early stages, *Sof-pax2/5/8* was detected in mantle, arms, funnel tube territories and gills but not in the funnel pouch territories (Fig 4I–4K). On the head, the covering tissue issued from arm pillars area expressed *Sof-pax2/5/8* (Figs 4K and 6I), as observed with *Sof-pax3/7*.

Expression of *Sof-pax2/5/8* in the central nervous structures was only detected during late development (from stage 23), restricted to the ventral side of the brain, in anterior and median suboesophageal masses (Fig 5F). The optic tractus (connecting the optic lobe and the supra oesophageal mass) transiently expressed *So-pax2/5/8* at stage 24 (Fig 5J). These expressions stopped by stage 25. In peripheral nervous system, *Sof-pax2/5/8* was expressed in the intrabrachial nervous cords from stage 23 (Fig 5I) and in stellate ganglia, at least between stages 21 and 24 (Fig 5K).

In arms, *Sof-pax2/5/8* was faintly detected in the epidermis at stage 19 (Fig 6F) but its later expression was clearly mesodermal (Fig 5I, Fig 6F and 6G). Staining intensity was asymmetric (anti oral side), different between the arms (stronger in the most ventral arm 5) and extended along the arms as they grew. A mesodermal *Sof-pax2/5/8* expression was also observed in the developing fins (Fig 6H). In the funnel tube a mesodermal signal was also obvious in stage 19–20 (Fig 6F and 6G) and was later restricted to the dermis (Fig 6K). The epithelial funnel organ was clearly stained (Fig 6K). By contrast, *Sof-pax2/5/8* expression was epidermal in the mantle at stage 19 (Fig 6E) and later extended to the dermal layers but not to the muscular layer of the mantle (Fig 6L). Finally, an expression in gills was observed from stage 16 to 24 in the epithelium, as for *pax6* (Fig 6E and 6J).

## Discussion

### An expanding repertoire of *Pax* genes

Our analysis confirms the existence of six *Pax* subfamilies including the Pax-α/β subfamily already proposed [11–13] and illustrated in our set by a strongly supported Pax-β group which is putatively specific to Lophotrochozoa. The origin of this subfamily is unclear and awaits further analysis. The roles of Paxβ remain largely unknown. Two isoforms *Hau-Paxβ1* and *Hau-Paxβ2* have been described in the leech *Helobdella*. *Paxβ1* is expressed during early cleavage stages in *Helobdella austinensis* embryogenesis and is probably implicated in the transition from spiralian to symetrical segmentation during spiralian development [28]. Both paralogs are also expressed at several different stages in segmental mesoderm (*Helobdella robusta* [12]), with unknown functions. Moreover, under the hypothesis that the *Biomphalaria glabrata* Paxβ-like is physiologically relevant, the absence of a PD suggests that these proteins may not always act as transcription factors as previously suggested for some *Pax* splice variants [15].

The Pox neuro subfamily is more common than previously described as it is found in all lophotrochozoan clades (this work) as well as in protostomian clades, hemichordates and echinoderms [11]. Currently, the function of the PoxNeuro protein has only been studied in *Drosophila*. It is known to play a role in the specification of neuronal identity in central and peripheral nervous system [67,68], in morphogenesis of appendages [69] and in the control of various aspects of male courtship behaviour [70]. This subfamily clearly deserves more attention.

Contrary to the pattern reported in chordate, the *Pax2/5/8* subfamily is devoid of HD in all accessible lophotrochozoan sequences; this fact should be taken into account in future proposals for the evolution history of *Pax* genes (e.g. [11]). Similarly, no HD was detected in *Octopus* Pax6 sequence which is quite surprising as complete *Pax6* are found in other cephalopods. Yoshida et al. [20] have related the numerous splicing isoforms found in *Idiosepius paradoxus* to the evolution abilities of cephalopods eye, but likewise the HD was clearly modified in three of the five *Pax6 Idiosepius* isoforms (first alpha-helix absent in *Pax6v1* and *Pax6v2*, alpha 3/4 helix disrupted in *Pax6v4* [71]). Analysis of mouse mutants has shown that *Pax6* HD plays an important role in eye development [72] but no role in the regulation of neurogenesis in the developing forebrain, although it is partially required together with the PRD for some—but not all—boundary formation in the forebrain [73]. *Pax6* HD is also dispensable for pancreas development [74]. The physiological significance of these short forms in Lophotrochozoa is currently unknown.

Finally, our analysis underlined misinterpretations in the databases (see Table 1 and S1 Table). We subscribe to the conclusion of Friedrich {Friedrich:2015fz} about the utility for the community to build a consolidated *Pax* homolog database.

### *Pax6* expression: Conserved in the brain and in eye tissues, but not in the retina

As already underlined in [33], *Sof-pax6* expression was largely distributed in central nervous system (CNS) areas. The role of *pax6* in the development of the CNS appears to be conserved in cephalopods. The expression of *Sof-pax6* in the brachial nervous chord suggests that the role of *pax6* in neural development is extended to this morphological novelty of cephalopods. Likewise, the restriction of Sof-pax6 expression at the distal tip of growing arms, which is described as a growing/proliferation region in *Octopus* [75] and *Euprymna* [76], suggests a role of Sof-pax6 in the growth of the arms.

*Pax*6 is considered as the master gene in eye development [77,78]. In *Drosophila*, homologs of *Pax*6 (*Ey* and *Toy*) are atop the retinal determination gene network (RDGN), a group of transcription factors and cofactors controlling eye development and including *Pax6*, *Sine oculis (So)*, *Dashchund* and *Eyes absent (Eya)* (review in [79]). The expression of *Pax6* in developing photoreceptors (i.e. retina) has been demonstrated in rhabdomeric [80] as well as in ciliary [35] photoreceptors in numerous metazoans, including Lophotrochozoa. Cephalopods have camerular eyes with rhabdomeric photoreceptors, but it is noteworthy that the role of *Pax6* in the development of retina seems far less obvious in cephalopods. In *S*. *officinalis*, *Sof-Pax6* was expressed from early to late development in cornea and tissues surrounding the eye but never in the retina cells throughout all stages of development (see Fig 5A and 5B). In addition, the probe used in our study covered a common sequence of *Sof-pax6* splice variants identified in a recent *S*. *officinalis* transcriptomic database (unpublished data), suggesting that none of them are expressed in retina. Likewise, Yoshida et al. [20] have shown that none of the six *Idiosepius Pax6* isoforms is expressed in the retina during development, questioning the paradigm of the *Pax*6-photoreceptor link. No other *Pax* gene (see below) seems to be involved in the organogenesis of the retina in *S*. *officinalis*. By contrast, the role of *otx2* in the early development of the retina is probably conserved in *S*. *officinalis* as its expression, in a restricted but unidentified set of cells (stem cells? future support cells?), is observed in retina [81].

The widespread expression of *Pax6* in developing eyes of metazoans has led to the hypothesis of a monophyletic origin of the structures dedicated to photoreception. The conservation of the genetic control of eye development by *Pax6* among all bilaterian animals would then not be due to functional constraints, rather, a consequence of its evolutionary history [78]. These ideas are considered as an oversimplified view by others [61,82], as some examples of eye developing without any *Pax6* expression are already known: developing *Limulus* eyes [83], developing *Platynereis* adult (but not larval) eyes [84], Hesse organs (eye cups) of amphioxus [85], eye regeneration in planarians [58] as well as formation of the Bolwig organ (larval eyes) in *Drosophila* [86]. The developing *Sepia officinalis* eye may be added to this list, at least for the main functional part in photoreception (i.e. retina). Nevertheless, *So-Pax6* seems to contribute to the formation of other parts of the visual organ. Studies of *Pax* expression in basal bilaterian have shown that other *Pax* classes can master eye formation: *PaxB* (*Pax2/5/8* subfamily) in the cubozoan jellyfish *Tripedalia* [87], *PaxA* (*PoxN* subfamily) in the hydrozoan *Cladonema radiatum* [88]. The fact that all these « master genes » belong to the *Pax* family is interpreted by Suga et al. [88] as a confirmation for the hypothesis of a monophyletic origin of photoreceptors. This argument is not validated by our current data regarding *Pax* gene expression in the retina of *S*. *officinalis* as we did not detect any other *Pax* expression at any stage. However, the hypothesis of a secondary loss of *Pax6* role in photoreceptor differentiation with a co-optation of *Pax1/9*, *Pox neuro* or *Paxβ*, cannot be yet discarded.

### *Pax3/7* expression: In ectodermal tissues but not in muscles

The involvement of the *Pax3/7* family in skeletal muscle development [89] is specific to vertebrates (and maybe nematodes [90]). *S*. *officinalis*, as other Lophotrochozoa, is not an exception since no expression was detected in any muscular tissues, including systemic heart. The expression of the *pax3/7-A* paralog is also mesodermal in the leech *Helobdella robusta* [19], and it is involved in nephridies development and body cavity formation. We did not observe any clear expression of *So-pax3/7* in mesodermal tissues.

In vertebrates, *pax3* and *pax7* also contribute to the development of the nervous system [62,91,92] and this role seems more largely conserved among Metazoa. Their homologs in *Drosophila* (*Gooseberry* and *Gooseberry-neuro* [7]) are essential segment-polarity genes [93] and are involved in neurogenesis [94]. This role in nervous system development seems conserved in *S*. *officinalis* as expression is conserved in brain. Nevertheless this neural expression is late, suggesting that *Pax3/7* is not involved in early neurogenesis (the emergence of ganglia), and restricted to the ventral brain in “motor” areas controlling the arms.

*Pax3/7* is expressed as longitudinal neuroectodermal bands in two annelids, *Platynereis dumerilii* [27] and *Capitella teleta* [23]. In *S*. *officinalis*, *Sof-pax3/7* is expressed in ectodermal tissues, such as the skin of the arm pillars covering the head. An involvement of *Pax*3/7 in the development of ectoderm is not surprising. In *Drosophila*, *Pax3/7* (*gsb*) specifies the epithelial pattern of each segment, including neuroblast specification [95]; in vertebrates, *Pax3* and *Pax7* regulate the development of neural crests at the origin of epidermal pigmentary cells [92,96]. The exact roles of Pax3/7 in cephalopod skin development, including the differentiation of several types of epidermal sensory cells, remains to be determined.

### Pax2/5/8 expression: In brain and mesodermal tissues but not in sensory organs

*Pax2/5/8* expression has been characterized from Lophotrochozoa belonging to Annelida [27,28] but its expression during development has only been followed in four molluscan species [21,31].

No clear *Pax2/5/8* expression could be observed in *S*. *officinalis* statocytes during organogenesis (from stage 16), as also shown by Wollesen et al. [21] in *Idiosepius notoides* (Cephalopoda) from stage 19. This differs from what is observed in other protostomes. In the developing (until 78hpf) mollusc *Haliotis asinina*, the statocyst and chemio / mechanosensory cells also express *Has-Pax2/5/8* [31] In *Drosophila* early embryos, *Pax2/5/8* is expressed in labial and antennal mechanosensory organs [97]. No expression of *Sof-pax2/5/8* has been detected in eyes whereas an expression has been detected in optic area of veliger larvae and in eye area after metamorphosis of *Haliotis asinina* [30,31] as well as in differentiating *Drosophila* omatidia [97]. Based on these results, we suggest that the role of Pax 2/5/8 in sensory structures development could have been lost in cephalopod lineage. Nevertheless, *Sof-pax2/5/8* transient expression in optic tractus suggests a role for *Sof-pax2/5/8* in the development of neuronal circuits for vision. A role of *Pax2* in the development of the optic chiasm and in the guidance of axons of the optic nerve has also been shown in vertebrates [98].

Quite unexpectedly, and unlike *Sof-Pax3/7*, *Sof-Pax2/5/8* was expressed in mesodermal structures. Early mesodermal expressions of *Sof-Pax2/5/8* in arms and funnel tube are remarkably similar. These observations are in agreement with the hypothesis of a common origin for arms and funnel tube, different from the funnel pouch [99] which does not express *Sof-Pax2/5/8*. Expression in mesodermal tissues has already been shown in the Annelida *Helobdella austinensis* where *Pax2/5/8* has a role in the symmetric cleavage of the mesodermal proteoblast [28]. Interestingly, *Pax2/5/8* is expressed in typical molluscan structures in *Haliotis asinina* larvae: dorsolateral cells of the foot, right shell muscle and in the pallial chamber [31]. The expression of *Sof-Pax2/5/8 in S*. *officinalis* arms and funnel, considered as derived from the molluscan foot, suggests a conserved role in derived structures and morphological novelties.

*Pax2/5/8* expression is maintained, at least in muscle, in the adult *Haliotis asinina* [30]. In *S*. *officinalis*, *Sof-pax2/5/8* might be involved in early steps of myogenesis in the locomotor structures derived from the foot during evolution, but is clearly not implicated in muscle differentiation. This hypothesis would imply the recruitment of other genes in the formation of muscles, particularly in the mantle. In this context, *NK4* is a good candidate [54]. On the other hand, the early expression of *Pax2/5/8* in the brain occurs in anterior and middle subœsophageal mass, derived from the pedal ganglia and known to control the arms and the funnel in the adult [63]. The development of *S*. *officinalis* is direct but the embryo is able to move and react inside the capsule from stage 24/25, implying a functional connection between muscular and nervous systems from these stages. *So-pax2/5/8* could be implicated in the formation of the whole nervous circuitry controlling arms and funnel muscles. In further development, *So-pax2/5/8* is expressed in nervous territories involved in motor control: intra-brachial nervous system, subœsophageal masses, stellate ganglia (through which pass the giant fibres innervating muscles). *Pax2/5/8* may thus play a major role in the neuro-muscular complex development.

Finally, *Pax2/5/8* expression in the gills of *S*. *officinalis* underlines an interesting and provocative problem. Expression of *Pax2/5/8* in gills has been detected in adult gastropoda [30]. It has also been recorded in urochordate *Oikopleura dioica* [57], amphioxus [100] as well as in furrows separating branchial arches in *Xenopus* embryos [101]. This leads to a discussion between a ‘ciliated placode formation’ versus a ‘perforation’ role for *Pax2/5/8* (see [57]). The gills in cephalopods emerge from a small cellular blastema; to our knowledge, they are neither considered as homolog to chordate gills nor ciliated. However, on the basis of the common expression of *Pax2/5/8* in “gills” from urochordate, cephalochordate, vertebrate and molluscs (cephalopods and gastropods), these structures may be taken as homologous (as defined by [42], see [102]) and should exist in Urbilateria. One possible alternative is that the biological functions governed by *Pax2/5/8* in this case are ancestral and used by analogous structures, even under the control of orthologous genes.

## Conclusion

The diversity of the *Pax* family is proposed to explain the high functional diversity of *Pax* proteins [10,22]. We observed overlapping of different *Pax* gene expression patterns in *S*. *officinalis*, although we cannot yet ensure that these observations correspond to a coexpression at the cellular level or only to a concomitant expression in neighbouring cells. Such overlapping of gene expression patterns has been observed for *Platynereis Pax3/7*, *Pax6* and *Pax2/5/8* in the nervous system, where it was interpreted as a “spatial code” defining longitudinal neural progenitor domains [27]. Alternatively, some overlapping areas between paralogs could be regarded as “redundancy islets” allowing further evolution. Functional redundancy between *Pax6* and *Pax2* has been demonstrated in mouse [103]. Moreover, *Pax* genes are known to be prone to abundant alternative splicing (e.g. [15,17,101]). The results of Short et al. [24] suggest that the functional network of co-expressed *Pax* isoforms is more evolutionary constrained than each of the isoforms, because orthologous isoforms, even if conserved during evolution, may individually have very different transcriptional activities in different species. Thus, functional studies are in fact needed to confirm inferences proposed on the basis of simple gene homology.

## Supporting information

S1 Primers primers for *Sof-pax* sequences. Location of primers used for probe synthesis. Green: forward primers; pink: reverse primers. The grey blocks correspond to PRD and OM (present only in Pax2/5/8 and Pax3/7 sequences) domains respectively. The HD domain is not presented.
(DOCX)

S1 Table List of lophotrochozoan *Pax* sequences. Accession: accession number or genome reference (DNA/RNA: corresponding sequence). Submitted name: name found in databases. Proposed name: our interpretation (bold type if different from submitted name). (f) denote a fragment. Please note that sequences from *Sabellaria alveolata* (SA_Locus_72760, SA_Locus_22824, SA_Locus_22463) are unpublished and courtesy of P-J. Lopez and J. Fournier, UMR BOREA (personal communication).
(XLSX)

S1 Alignment Lophotrochozoan *Pax* alignment. Alignment of the sequences given in S1_Table.xlsx, in fasta format.
(FAS)
