# Supplementary material for: The Pax gene family: Highlights from cephalopods
Source: PLoS One. 2017 Mar 2;12(3):e0172719. doi: 10.1371/journal.pone.0172719 (PMC5333810; doi:10.1371/journal.pone.0172719)
Supplement: S1 Primers — Location of primers used for probe synthesis. Green: forward primers; pink: reverse primers. The grey blocks correspond to PRD and OM (present only in Pax2/5/8 and Pax3/7 sequences) domains respectively. The HD domain is not presented. (DOCX) [file pone.0172719.s001.docx]

Supporting information S1 : Primers

....|....| ....|....| ....|....| ....|....| ....|....| ....|....| ....|....| ....|....| ....|....| ....|....|

5 15 25 35 45 55 65 75 85 95

Pax2/5/8|KP867644 ATGGACCTAA CCACAGCGTA CCGTTACCAC AACACTAATC TAAATATGAT GGACTTCTAT CACTCTTGTA AAATGCTCAA TGGCTCCAAA CATAATGAAA

Pax3/7|KF739402 ATGG.T T.CC.AC..C

Pax6|AM422131

....|....| ....|....| ....|....| ....|....| ....|....| ....|....| ....|....| ....|....| ....|....| ....|....|

105 115 125 135 145 155 165 175 185 195

Pax2/5/8|KP867644 CCTTTACAGG ATTTTATGAA GCAACAGAAG GCCACGGCGG AGTTAACCAA CTAGGTGGTG TGTTTGTTAA CGGGAGGCCC TTACCGGACG CAGTTCGGAC

Pax3/7|KF739402 GAAAGGAG.A CAA.CG.TCG CTT.T.A... ....G..AA. ...A...... ..G..A..G. .A...A.C.. T..A.....G C.C..AA.TC AT......CT

Pax6|AM422131

....|....| ....|....| ....|....| ....|....| ....|....| ....|....| ....|....| ....|....| ....|....| ....|....|

205 215 225 235 245 255 265 275 285 295

Pax2/5/8|KP867644 GCGTATTGTG GAATTGGCCC ACCAAGGGGT CCGACCATGT GATATCTCTC GACAGTTACG TGTCTCCCAT GGTTGCGTCA GTAAAATTCT GGGCAGGTAT

Pax3/7|KF739402 TAAA..C..T ...C....AG CG........ T.....C... .TG...AGC. .C..AC.G.. G..G..T..C ..A....... .......C.. CCAGC.C..C

Pax6|AM422131 ..... C..AC....C

....|....| ....|....| ....|....| ....|....| ....|....| ....|....| ....|....| ....|....| ....|....| ....|....|

305 315 325 335 345 355 365 375 385 395

Pax2/5/8|KP867644 TGTGAAACTG GGTCTATAAA ACCAGGCGTA ATCGGCGGTT CGAAGCCCAA AGTAGCCACC CCAAAAGTTG TAGAGGCCAT TTCAAAATAT AAGCAAGAAA

Pax3/7|KF739402 CAG.....C. .TAG....CG G..G..A..C .....G..C. ....A..GCG C..T..T..T ..TG.G..C. AGA..CGG.. CGAGC.G..C ...A....T.

Pax6|AM422131 .A...G..G. .C..C....G G..GC...CG .....G..CA GC.....A.G ...G..G... ..TG...... .GC..AAA.. AG.CC...TC ...AGG..GT

....|....| ....|....| ....|....| ....|....| ....|....| ....|....| ....|....| ....|....| ....|....| ....|....|

405 415 425 435 445 455 465 475 485 495

Pax2/5/8|KP867644 ATCCGACCAT GTTTGCCTGG GAGATTCGCG ATCGTTTATT ATCAGAGAAT ATCTGCAGCC AAGACAATGT TCCGAGCGTC AGTTCTATTA ACAGAATAGT

Pax3/7|KF739402 .C..AGGT.. T..CAG.... .......... .CAAGC.GC. GAA...AGGC G.G...GA.. GCAG..CG.. G..C...... .....C..C. GTC.TG.GC.

Pax6|AM422131 GC..CT.G.. A..C..A... ..A...A.G. .C..A..GC. G.....AGGA G.T....CA. .G..T...A. A..A..T..T TC...G..A. ....GG.GC.

....|....| ....|....| ....|....| ....|....| ....|....| ....|....| ....|....| ....|....| ....|....| ....|....|

505 515 525 535 545 555 565 575 585 595

Pax2/5/8|KP867644 CCGGAATCGA GCCGCAGAAA AAGCCAAGGC TCAAAACCCG CAGGCCCAGT CGAGTCCCCC ACTTCAAGGT GAGGCCCCAC CACCGGCTCT TAACCAGACT

Pax3/7|KF739402 .A...GC..G TTTCACAGCG ...A.G.T.A CG.TG.TTGC G.C.A.G.CG ACGA.GA.GA .AAA.GGATC A.AATGG.T. GCAGT.AGAA CGGAAGCGAC

Pax6|AM422131 T..T....TT ..AAGC.... .TCAG....T ..TTGGT.A. GG.A.TACAA T.TACGATAA ....GGTCT. CT.AATGGT. A.G.TTGG.C ACG..CA.A.

....|....| ....|....| ....|....| ....|....| ....|....| ....|....| ....|....| ....|....| ....|....| ....|....|

605 615 625 635 645 655 665 675 685 695

Pax2/5/8|KP867644 CAGGCTCCTG CTGGGGCAGA TGGTATGACA CGACCCGGAC CCTATTCAAT AAGTGGAATC TTGGGAATCC CTTCTCAAAA CTCAGCGAAT TTGAATAATG

Pax3/7|KF739402 A.AAA.A.CA ACCAC AGC.. CGAC.....T ..A...GAGG TCAAA.TGC. .AA.T.CG.C AAAG..G..A

Pax6|AM422131 .CCTGGTAC. .ACCTAAT.C CTCC...

....|....| ....|....| ....|....| ....|

705 715 725 735

Pax2/5/8|KP867644 GAAACTTTAA ACGGAAAAGA GATGACGAAT CGGTG

Pax3/7|KF739402 TCTCAGACGC TGACTCGGA. CCG.GATTTC AA..C

Pax6|AM422131
